# *Trichoderma* and the Plant Heritable Priming Responses

**DOI:** 10.3390/jof7040318

**Published:** 2021-04-19

**Authors:** María E. Morán-Diez, Ángel Emilio Martínez de Alba, M. Belén Rubio, Rosa Hermosa, Enrique Monte

**Affiliations:** Department of Microbiology and Genetics, Spanish-Portuguese Institute for Agricultural Research (CIALE), University of Salamanca, Villamayor, 37185 Salamanca, Spain; aemarti@usal.es (Á.E.M.d.A.); belenru@usal.es (M.B.R.); rhp@usal.es (R.H.); emv@usal.es (E.M.)

**Keywords:** biocontrol, systemic defence, immune response, epigenetics, methylation, transcription factor, inheritance

## Abstract

There is no doubt that *Trichoderma* is an inhabitant of the rhizosphere that plays an important role in how plants interact with the environment. Beyond the production of cell wall degrading enzymes and metabolites, *Trichoderma* spp. can protect plants by inducing faster and stronger immune responses, a mechanism known as priming, which involves enhanced accumulation of dormant cellular proteins that function in intracellular signal amplification. One example of these proteins is the mitogen-activated protein kinases (MAPK) that are triggered by the rise of cytosolic calcium levels and cellular redox changes following a stressful challenge. Transcription factors such as WRKYs, MYBs, and MYCs, play important roles in priming as they act as regulatory nodes in the transcriptional network of systemic defence after stress recognition. In terms of long-lasting priming, *Trichoderma* spp. may be involved in plants epigenetic regulation through histone modifications and replacements, DNA (hypo)methylation, and RNA-directed DNA methylation (RdDM). Inheritance of these epigenetic marks for enhanced resistance and growth promotion, without compromising the level of resistance of the plant’s offspring to abiotic or biotic stresses, seems to be an interesting path to be fully explored.

## 1. Introduction

*Trichoderma* is one of the most studied genera of ascomycetous fungi because of the importance of the practical applications and particular skills of the species that compose it [1,2]. Possibly, its main characteristic is the ability to exert positive effects on plants by means of the production of cell wall degrading enzymes (CWDE) [3] and metabolites with antimicrobial activity [4], and the release of volatile organic compounds (VOC) [5,6], which can act together as direct biocontrol agents on phytopathogenic fungi, oomycetes, nematodes, and bacteria [7]. *Trichoderma* spp. also compete with other plant beneficial microbes and impact the arbuscular mycorrhizal fungi (AMF) abundance in bulk soil microbiomes in agricultural systems [8]. In this sense, a single application of *T. harzianum* increased the rhizosphere levels and facilitated the access of AMF to the roots of the host (e.g., wheat) [8] and non-host (e.g., rapeseed, *Arabidopsis*) [9] plants. *Trichoderma* spp. can also act as indirect biocontrol agents by activating systemic immune responses in a coordinated way [10], resulting in faster and stronger induction of plant basal resistance mechanisms upon the perception of a later triggering stimulus. This phenomenon is known as priming of defence [11] and *Trichoderma* can provide the plant with long-lasting resistance against biotic and abiotic stresses by balancing the different phytohormone-dependent pathways—among which salicylic acid (SA), jasmonates (JA), ethylene (ET), abscisic acid (ABA), auxin (indole-3-acetic acid: IAA), and gibberellins (GA) are the most relevant—and modulating the levels of growth and defence regulatory proteins [12]. The advantage to the plant being primed for particular stress responses is in facilitating a faster and stronger reaction if the stress recurs [13]. Reinforced responses to pathogen attacks come under the category of induced defence, while responses to abiotic stresses are referred to as acclimation or hardening, even though these responses are very similar in their genesis and can also be enhanced by priming treatments [13]. An accurate definition of how *Trichoderma* exerts its beneficial action on plants is of particular relevance to the way in which commercial products based on the abilities of *Trichoderma* are registered.

Regardless of whether *Trichoderma* is recognized by the plant through cell-surface pattern recognition receptors (PRRs), priming involves multiple cellular localizations of targets such as: (i) inactive metabolites stored in vacuoles, which can be hydrolysed and released into the cytoplasm; (ii) reactive oxygen species (ROS) that can also interact with phytohormone-dependent pathways in the cytoplasm; and (iii) epigenetic changes that occur in the nucleus [14]. Cascades of mitogen-activated protein kinases (MAPK) and Ca^2+^-dependent protein kinases (CDPK, especially related to plant growth regulation and acclimation, [15]) function downstream of the ligand binding and the subsequent PRR activation and transmit extracellular priming stimuli into intracellular responses, while amplifying at the same time the transduction signals to the nucleus, resulting in transcriptional reprogramming, callose deposition, and, ultimately, synthesis of hormones and activation of defence-related genes [16].

Priming involves enhanced accumulation of dormant cellular proteins functioning in intracellular signal amplification [11,17], as is the case with: (i) signalling proteins in an inactive configuration that are activated upon exposure to stress, as occurs with MAPKs being triggered by the rise of cytosolic Ca^2+^ levels and cellular redox changes following a stressful challenge; (ii) transcription factors (TF) (e.g., auxin response factors (ARF), ethylene response factors (ERF), MYBs, MYCs, WRKYs) acting as regulatory nodes in the transcriptional network of systemic defence after stress recognition; and (iii) epigenetic regulation involving histone modifications and replacements, DNA (de)methylation, and RNA-directed DNA methylation (RdDM) [13,14,16,17,18,19,20].

In plant–*Trichoderma* systems, the actions of one trigger counteract the other. We refer to a dynamic process, which follows its own zig-zag defence model [1], in which four timing stages might be identified—Stage 1: the plant’s early perception of *Trichoderma* that only lasts a few hours; Stage 2: cell signalling, giving rise to systemic defence responses that can reach several days, gradually decaying in weeks; Stage 3: long-lasting priming; Stage 4: transgenerational inheritance. The latter two stages last from a few weeks to the offspring stage. Here, based on our own and other authors’ experimental evidence, we want to present how plants respond to the stimuli caused by *Trichoderma*, stressing that such responses are transmitted onto the progeny, in terms of different types of defence, acclimation, and growth control.

## 2. Plant’s Early Perception of *Trichoderma*

**Figure 1:**
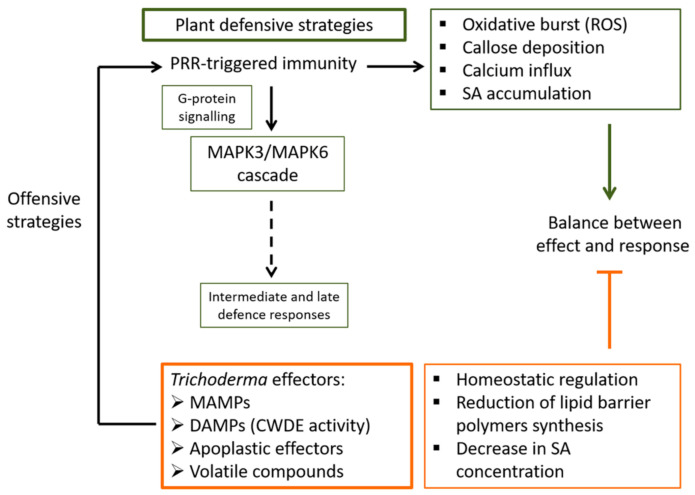
Schematic outline of plant’s early response to the interaction with *Trichoderma.* Plant´s cell surface pattern recognition receptors (PRR) are the first line of defence against *Trichoderma* effectors during the early response (this first stage of the colonization being considered by the plant as an attack). As a result of this first encounter between the plant and *Trichoderma*, a series of events will be triggered in the plant including the rapid release of reactive oxygen species (ROS), the deposition of callose, calcium influx, and accumulation of salicylic acid (SA) at the primary inoculation site as well as for systemic defence. Another one of the early events triggered by PRR stimulation is the activation of the mitogen-activated protein kinases (MAPK) cascade mediated by the G-protein complex, which will lead to several intermediate and late defence responses. *Trichoderma* spp. will find their way to balance this early plant response into one of mutual benefit by regulating homeostasis and reducing the synthesis of plant lipid barrier polymers, as well as by decreasing the production of SA.

It has been observed that the root endospheric microbial community is less diverse than that of the rhizosphere, which indicates that many fungi in the rhizosphere might be trying to enter the plant, but only a few among them are allowed and prove successful [8,21]. This is the case for *Trichoderma* that manages to use the needed mechanisms in order to achieve its aims. *Trichoderma* activates PRRs by means of microbe- or damage-associated molecular patterns (MAMP or DAMP), apoplastic effectors, and a variety of VOCs [12,22,23,24]. Then, oxidative burst, callose deposition, and Ca^2+^ and ROS signalling are initiated rapidly following the attack because, at that point in its perception of *Trichoderma*, the plant does not recognize that it is a friendly attack. Secreted *Trichoderma* molecules are sensed by plant cells through intracellular Ca^2+^ changes that lead to not only intracellular ROS accumulation but to the necessary adjustments to prime defence mechanisms [25]. Figure 1 summarizes some of the main effects and responses triggered during the early interaction between the plant and *Trichoderma*. It is well established that G-proteins located within the cell membrane can be the linkage between host-derived signals and *Trichoderma*-intracellular signalling pathways, resulting in an increase in mycoparasitism-relevant processes such as the production of fungal CWDEs and antifungal metabolites, and the formation of infection structures [26]. Although the activation of G-protein signalling is different in animals and fungi than in plants, it can be thought that G-proteins may play a role in the recognition of *Trichoderma* by the plant. In this way, heterotrimeric G-proteins function as a convergence point of plant defence signalling by mediating responses initiated by the activation of different PRRs, as is the case of systemically transmitted stomatal closure, ROS production, or callose deposition at the cell wall [27]. There is also evidence for the role of G-protein-dependent regulation downstream of PRRs in plant growth and development [28], which indicates a molecular mechanism of G-protein recognition and signalling activation/deactivation compatible with the effects that *Trichoderma* has on plants. This fact constitutes a promising line of research to understand the initial dialogue between *Trichoderma* and the plant.

Most of the work on *Trichoderma*–plant interactions has been carried out in root systems. However, it should not be forgotten that many *Trichoderma* strains live and can be isolated from the aboveground parts of the plant [29], where they endophytically colonize leaves and stems [30]. Some strains of *Trichoderma* are effective in the direct control of pathogens in the phyllosphere, although their mechanism of action seems to be linked more to induced resistance and mycoparasitism than to direct competition [31]. Selected *Trichoderma* strains may also release VOCs related to plant growth promotion [32] but may also be involved in reducing energy losses and inducing photosynthesis and resistance to foliar pathogens, such as those producing powdery mildew [33]. Even endophytic *Trichoderma* strains can mitigate the negative impact of leaf-cutting ants in both agriculture and silviculture due to the potential to act as bodyguards to their plant hosts affording protection to the whole plant [34].

ROS production is one of the earliest responses, starting only a few minutes after the PRRs switch on, although, it also occurs later when the defence is already established, but at a much slower pace. ROS have been proposed to act as antimicrobials, as cross-linkers of the plant cell wall to block pathogen entry, and as local and systemic secondary messengers to trigger additional immune and developmental responses [35]. When colonizing the roots, *Trichoderma* spp. go beyond the oxidative burst generated as an early defence response by the plant. So, *Trichoderma* strains take advantage of their ability to compete in the rhizosphere, since they have the particularity of tolerating high levels of ROS [36] and even produce ROS by themselves, which in return facilitates the biocontrol activity against pathogens, most of them with a lower range of oxidative stress tolerance [37]. The establishment of *Trichoderma* in the root tissues may be fostered by the action of plant CWDEs such as hemicellulases and polygalacturonases. In *T. harzianum*, endopolygalacturonase activity was required for active root colonization and the release of oligogalacturonides, oligomers of α-1,4-linked galacturonic acid generated by the partial hydrolysis of pectin, capable of acting as DAMPs to activate the systemic defences against the necrotrophic leaf pathogenic fungus *Botrytis cinerea* [38]. During the colonization of the root, SA is the key phytohormone to reinforce plant cell walls, restricting *Trichoderma* to the apoplast and preventing the arrival to the vascular system. The important role of SA in *Trichoderma* root colonization was demonstrated with *Arabidopsis sid2* mutants unable to synthesize SA, as plants lacking this phytohormone did not accumulate callose and allowed the dissemination of *Trichoderma* in the vascular ring and the upward movement to the aboveground part of the plant, which would lead to a subsequent collapse [39]. Therefore, it seems clear that *Trichoderma* has to cope with the oxidative burst, callose deposition, and SA-signalized immunity during the first hours of the interaction, when the early SA-dependent defence response may not reach its full potential. This struggle allows *Trichoderma* to colonize the root, although the down-regulation of SA would reach a balanced level where there is no indiscriminate spread of the fungus [22]. Thus, one of the first tasks *Trichoderma* needs to do is to accurately overcome and counteract the plant’s initial defences. In this regard, we have observed a strong down-regulation of the SA-responsive *PATHOGENESIS-RELATED* (PR) *PROTEIN 1* (*PR-1*) gene after 6 h of cultivating *Arabidopsis* in the presence of *T. harzianum* [40], and it has also been reported as early as after 4 h of *Arabidopsis*–*Trichoderma* interaction, as a strong down-regulation of the SA-related *ENHANCED DISEASE SUSCEPTIBILITY 1* (*EDS1*) gene. EDS1 operates upstream of SA-dependent defences and is required early to accumulate SA, although it is also required later to generate the plant hypersensitive response (HR) to prevent the spread of infection by microbial pathogens [41]. SA production is not the only defence mechanism limited early on by the effect of *Trichoderma*. Syntheses of lipid barrier polymers and signalling during pathogen attack [42] are down-regulated in *Arabidopsis* root tissues 24 h post inoculation (hpi) with *T. harzianum* [40]. In this way, the rapid and local silencing of early plant defence response enables *Trichoderma* to establish symbiotic interactions, as it has been observed for other symbiotic systems [43]. The down-regulation of PR proteins in maize seedling roots treated with *T. afroharzianum* is in agreement with the local silencing of defence genes to allow fungal growth into the roots [44]. However, following the decrease of defences in *Arabidopsis* root colonized by *Trichoderma*, the expression of both *PR-1* and *EDS1* genes was increased as early as 48 h [40]. In addition, the accumulation of PR proteins and catalase (CAT) activity increased in both the roots and leaves of cucumber seedlings at 48 hpi with *T. asperelloides* T203 [45]. Corroborating this fact, it has also been described that tomato plants showed a sharped increase in the expression of the *PR-1b1* gene at 72 hpi with *T. longibrachiatum* [46].

Based on the assumptions that each plant–*Trichoderma* interaction is very particular, that different activations of defence-related genes occur among *Trichoderma* strains against the same pathogen [47], and that it is difficult to establish a specific time point when early defence responses end, it has been proposed that 48 hpi would indicate the moment of transition when the plant reprograms its transcriptional machinery mainly towards redox and defence processes, fully accepting that *Trichoderma* is not an enemy [48]. There is no doubt that *Trichoderma* needs to minimize levels of SA-dependent defence responses—or at least prevent them from rising—but the maintenance of certain levels of SA is also very important in the establishment of an early oxidative burst. ROS generation, including superoxide anions and H_2_O_2_, is indicative of early defence response activation since they do not only act directly as toxic agents against the host plant cells but also against the pathogens by killing them or ceasing their virulent activity [49]. SA increases the accumulation of H_2_O_2_ in plants after pathogen infection. However, as observed in the first 24–48 h of tomato–*T. erinaceum* interaction, plants challenged with *Trichoderma* accumulated H_2_O_2_ at lower levels than those detected in plants infected with the pathogen *Fusarium oxysporum* f. sp. *lycopersici* (FOL) [50]. Similarly, a marked reduction in the H_2_O_2_ levels during the first 3–24 h of interaction between tomato plants and *T. asperellum* allowed the explanation of the protective effects exerted by *Trichoderma* against *F. oxysporum* and *B. cinerea* HR-like lesions [51]. These two cases may serve to illustrate that the plant accepts *Trichoderma* better than the pathogen.

Nevertheless, SA also plays opposite roles in preventing the damage to plants against oxidative stress by inducing the production of detoxifying enzymes such as CAT, superoxide dismutase (SOD), ascorbate peroxidase (APx), or glutathione S-transferase (GST), which reduce the accumulation of ROS as these molecules are not positive for plant cells either. We have observed that a *GST* gene strongly induced by SA, which participates in ROS detoxification, reinforcement of cell walls, formation of phytoalexins, and degradation of various toxic substances [52], was down-regulated in *Arabidopsis* plants at 24 hpi with *T. harzianum* [40]. In this sense, the treatment of *Arabidopsis* roots with *T. harzianum* activated, in just 4 h, the expression of the *CYP71A12* gene encoding a cytochrome P450 required in the biosynthesis of phytoalexins, reached the maximum peak of expression at 24 hpi but experienced a subsequent drop after that point [53]. In the tomato–*T. erinaceum* study, the CAT and SOD enzymatic activities were higher in primed plants than in those unprimed with *Trichoderma* under FOL challenged conditions [50]. In addition, SOD rose from 24 to 48 h with a drop to 72 h, while CAT dropped to 48 h and rose sharply to 72 h. A maize–*T. guizhouense* study [54] found that H_2_O_2_ was accumulated in maize tissues at 24 hpi in the interaction with *Trichoderma*. Additionally, the expression of the auxin synthesis related gene *YUC4*, was down-regulated at 12 hpi and at 48 hpi, with a peak of expression at 24 hpi, which indicates a decline in SA-dependent defences and lateral root growth. The examples of the undulating behaviour of plant responses given here can be explained by *SOD* gene up-regulation resulting in diminished ROS activity and therefore higher accumulation of H_2_O_2_, which in turn, induces subsequent CAT activity. After the initial oxidative burst, plant performance would be justified because ROS also functions at longer times as signalling regulators of growth and mitigation of biotic and abiotic stresses. If to all this we add that *Trichoderma* produces its own ROS to compete with other inhabitants of the rhizosphere [37], it would seem clear that *Trichoderma* works by fine-tuning the levels of ROS in the plant.

## 3. Systemic Plant Responses to *Trichoderma*

Stage 2 does not have a fixed starting time but depending on the system under study, it is considered not to start after 48–72 h of *Trichoderma* being applied. Upon root colonization, the plant produces a phytohormone blend (as a result of a series of transcription cascades that mainly involve SA, JA, and ET but also ABA, IAA, and GA signalling pathways) that constitutes the *Trichoderma* signal signature [10]. The signalling pathways that are activated by the endogenous accumulation of these phytohormone signals not only regulate the plant’s dialogue with *Trichoderma* but also contribute to deploying different active defence responses that are effective against distinct classes of attackers [55]. The effectiveness of the contribution of *Trichoderma* to protecting plants will depend on how the pathogens may have evolved to manipulate plants for their own benefit, interfering with the plant’s defence-signalling network [47]. At 72 hpi, changes in SA-induced defences may contribute to hindering further *Trichoderma* penetration but may also be indicating induced priming against other attackers [48]. Once the colonization has begun, the *Trichoderma* growth inside roots is restricted to the intercellular spaces of the cortex and epidermis, because callose deposition prevents access to the vascular bundles [45]. The endophytic accumulation of *Trichoderma* will also act as a physical barrier to prevent the movement of the pathogen in the plant [56]. *Trichoderma* strains from different species can colonize the rhizosphere and grow within the root tissue not only without causing damage but while priming plants against biotic and abiotic stresses [2].

It has been observed that upon challenge exposure to stress stimuli, elevated accumulation of mRNA transcripts and inactive MAPK proteins, such as MPK3, primes *Arabidopsis* plants for enhanced defence and provokes immunity to future invasions, whereas their down-regulation reduces immunity [57]. In this sense, the plant defence mediated by the activation of kinases MPK3 and MPK6 rapidly alters the expression of photosynthesis-related genes and photosynthetic activity, which promotes the accumulation of ROS and accelerates HR cell death [58]. The dual functionality of the MPK3/6 cascade in promoting defence and inhibiting photosynthesis potentially allows it to orchestrate the trade-off between plant growth and defence in plant immunity. Interestingly, *Agrobacterium tumefaciens* hijacks the MPK3/6 pathway for delivering the T-DNA into the plant cell nucleus [59]. MPK4 is another regulatory hub that is essential for basal resistance to invasive biotrophic and hemibiotrophic attackers that has a role in ROS signalling and negatively regulates the positive effects of EDS1 on SA-mediated defence, while also displaying positive regulation of JA/ET-dependent immunity [60].

In plant–*Trichoderma* interactions, root colonization was accompanied by the systemic expression of an *MPK3* orthologous gene in cucumber plants challenged with *T. asperelloides* and the overexpression of *MPK3* primed them for JA- and ET-mediated defences against *P. syringae* pv. *lachrymans* without previous root colonization by the beneficial fungus [61]. It was found in *Arabidopsis* that plant growth regulators, such as ET and IAA, produced by the plant in response to *T. atroviride*, control several aspects of the root growth but, simultaneously, they can modulate MPK6 activity affecting root development [62]. MPK6 also activates ET biosynthesis through the phosphorylation of 1-AMINOCYCLOPROPANE-1-CARBOXYLATE (ACC) synthase after PRR stimulation [63]. It was observed that after *T. viride* inoculation onto both sides of *Arabidopsis* leaves, MPK6 increased the expression of a plasma membrane proton ATPase, which had a regulatory role in further leaf colonization and plant growth promotion [32]. The MPK3/6 cascade regulates phytoalexin biosynthesis and the phosphorylation of the TF ERF6, triggering the ET signalling-dependent defences [64]. The MPK3/6 cascade leads to the activation of WRKY type TFs, such as WRKY33, culminating in the expression of defence genes. In this way, early transcriptional responses mediated by WRKY33 result in the down-regulation of SA-related plant defences, but also in the up-regulation of JA-associated responses at later stages [65], as reported in bean plants colonized with *T. velutinum* [66]. A summary of the TFs that have been reported to be relevant to the *Trichoderma*–plant interaction and their regulatory effect are presented in Table 1. Studies performed on *Arabidopsis* showed that bacterial infection resulted in increased accumulation of the metabolite azelaic acid (AZA) that has been shown to prime plants to accumulate higher levels of SA and the activation of systemic defence against the pathogen *P. syringae* [67]. AZA induces the expression of the *AZELAIC ACID INDUCED 1* (*AZI1*) gene, which encodes the secreted lipid transfer protein AZI1, involved in the production and/or translocation of a mobile immune signal [68], and has proven to be one of the targets of MPK3 and MPK6 [69]. All these cases may be indicative that signal transduction by the MPK3/6 cascade is not only necessary to induce defence responses but also constitutes a molecular node that fine-tunes the phytohormone networking that gives rise to a balance between growth and defence. The quick activation of MPK4, that negatively regulates SA accumulation and which later on regulates the inhibitory crosstalk between the SA and JA/ET signalling networks, is in agreement with the rapid (4 h) down-regulation of *EDS1* in tomato plants and the subsequent up-regulation of this gene at 48 hpi with *T. harzianum* [40]. The already indicated undulating responses of plants undoubtedly condition the goal of building up an effective systemic defence in the medium and long term.

In addition to the role of MPKs in the activation of WRKY33 within the nucleus, there are several examples where *Trichoderma* spp. are described as priming activators by modulating the expression of different TFs involved in transcriptional activation or repression at the end of the MAPK signal transduction cascades, resulting in accelerated activation of the defence responses followed by their subsequent moderation, which gives rise to the timing stages that lead to the different types of systemic resistance to stresses [47,70,71,72,73,74,75]. This *Trichoderma* trait is corroborated by the fact that cis-elements, known to be involved in stress responses, such as WRKY, MYB, and MYC motifs, have been found in promoters of secreted *Trichoderma* effectors [76,77].

TFs with highly conserved WRKY domains are quite common in plants (e.g., 38 genes in the moss *Physcomitrella*, 83 in tomato, and 197 in soybean) and play roles in the regulation of transcriptional reprogramming associated with plant stress responses against both biotic and abiotic factors. Genes induced during defence responses often contain WRKY TF-binding sites since WRKYs can interact (in)directly with MAMPs/DAMPs or effector proteins to activate or repress plant defences. In addition to the WRKY33 seen above being associated, respectively, with lowering and raising SA- and JA-dependent defences, experimental data pointed out the existence of crosstalk between induced defence and acclimation responses mediated by WRKYs [82]. The ankyrin repeat protein NONEXPRESSOR OF PATHOGENESIS-RELATED GENES 1 (NPR1) is a transcription cofactor that acts as the master key to the plant defence signalling network and mediates crosstalk between the SA and JA/ET responses [68]. 

*WRKY54* and *WRKY70* are direct transcriptional targets of NPR1 to negatively control SA accumulation [83]. WRKY54 and WRKY70 play dual roles in systemic defence, both as negative regulators of SA biosynthesis and as positive regulators of SA signalling. In a 24-h *Arabidopsis* root interaction with *T. harzianum* [40] and *T. asperelloides* [72], *WRKY54* was down-regulated. *WRKY54* and *WRKY70* were also down-regulated in *Arabidopsis* plants at 24 and 48 hpi with *T. atroviride*, with slight gene expression increases from 48 h until the relationship between *Arabidopsis* and *Trichoderma* was stably established after six days [73]. These results confirmed the important role of NPR1 as a negative regulator of SA biosynthesis in plants challenged with *Trichoderma* but also as an SA signal transducer at longer times. The structurally related WRKY18, WRKY40, and WRKY60 get accumulated in response to SA and JA during biotic stress as well as to ABA during abiotic stress responses. Interestingly, these TFs have leucine zipper motifs at their N termini via which they directly bind to their own respective promoters, in order to negatively regulate their expression patterns to maintain the homeostasis of WRKY proteins, which implies an additional element to understanding the crosstalk between *Trichoderma* and plants. WRKY18, WRKY40, and WRKY60 are also activators of T-DNA genes after their integration in the plant genome following *A. tumefaciens* infection [84], which also links them to the regulatory role of MPK3/6 in balancing defence and growth and opens a debate on how the use of T-DNA genes can affect the regulatory systems of plants transformed with the Ti plasmid. In *Arabidopsis* plants treated with *T. asperelloides*, the expression of *WRKY18* and *WRKY40* was enhanced at 9 hpi, which correlated with the negative regulation of the SA-dependent defence positive regulators *FLAVIN MONOOXYGENASE 1* (*FMO1*) and *EDS1* at the same time point. The activation of JA-signalling genes via the suppression of JASMONATE ZIM DOMAIN (JAZ) repressors was also observed [72]. The progressive decline in the expression levels of *WRKY18* and *WRKY40* reinforces the undulating behaviour of the defence responses in *Trichoderma*-colonized plants. To cite just one example of this pattern, *WRKY60* kept expression levels low in the first hours of plant–*Trichoderma* interaction and raised them after six days [72,73]. *WRKY4* gene expression is induced by SA and pathogen infection, and functions as a suppressor of SA-dependent defence genes. It was down-regulated in the first 24–48 h of inoculation of *T. erinaceum* in tomato [50] and *T. asperelloides* in *Arabidopsis* [72], although the levels of expression of *WRKY4* tended to increase over time. WRKY activation is not exclusive to MAPK cascades as it has been proven that CDPKs phosphorylate WRKY8 for immune gene expression and ROS production [85]. *WRKY8* in fact was strongly up-regulated at 24 h of *Arabidopsis*-*T. atroviride* interaction and presented a gradual decrease in its expression from then on [73]. A special point of attention is the case of *WRKY70*, which is a primary target for the *Arabidopsis* histone methylase ARABIDOPSIS TRITHORAX-LIKE FACTOR 1 (ATX1) involved in establishing the trimethylation pattern of lysine 4 (K4) in histone H3 (H3K4me3), while the SA-responsive gene *PR-1* and the JA-responsive gene *THI2.1* are secondary targets. The finding that the *PR-1* and *THI2.1* nucleosomes carry H3K4me3-marks unrelated to their transcription states has suggested that SA- and JA-inducible defence genes keep their nucleosomes in an ‘active memory’ state in preparation for quick-changes of transcription when needed by the cell [86]. As we will see later, after an initial stimulus, chromatin remodelling is stored in the defence gene promoters, which can be activated when plants are subjected to new stresses [87]. Similarly, histone methylations at the *WRKY40* promoter activate the SA-dependent pathway and negatively regulate ABA-dependent adaptation of plants to unfavourable environmental conditions [88]. This behaviour would be in accordance with what has been reported for *Arabidopsis*-*T. asperelloides* [72] and with the proposed plant–*Trichoderma* interaction model [10]. 

The R2R3-MYB TFs that only exist in terrestrial plants are capable of binding to the *WRKY* promoters leading to their expression (e.g., MYB44 directly regulates the expression of *WRKY70*; and MYB51 that of *WRKY33*) [89,90]. MYBs are involved in multiple stress responses, including defence-induced lignification and basal immunity (MYB15), SA-and JA-mediated defence responses and enhancement of ABA signalling (MYB44), indolic glucosinolate biosynthesis upon SA and ET signalling in *Arabidopsis* and salt/osmotic stress regulation (MYB51), early signalling of rhizosphere-induced systemic defence (MYB72), and auxin modulation and root development through interaction with ARFs (MYB77) [89,90,91,92,93]. The root-specific TF MYB72 is required for JA/ET-dependent systemic resistance and callose accumulation induced by *T. asperellum* in *Arabidopsis* plants and also functions as an early node of convergence in the signalling pathways that are induced by different beneficial microorganisms, such as *Trichoderma* itself and *Pseudomonas fluorescens* [70]. In *Arabidopsis* roots colonized by *T. asperelloides* [72], an undulating expression pattern was observed in different *MYB* genes, with maximum levels for *MYB15*, *MYB51*, and *MYB72* at 24 h. At this time point, the growth-regulating *MYB77* gene showed its minimal expression, limiting lateral root development while increasing defences, which would be corroborating the ability of *Trichoderma* to balance the costs of plant growth and defence [12].

BASIC HELIX-LOOP-HELIX (bHLH) MYC TFs regulate multiple functions in plants. In particular, MYC2 is required for JA-dependent systemic defences triggered by beneficial soil microbes, JA-regulated plant development, and lateral root formation. MYC2 acts as a regulatory hub within the JA signalling pathway and functions as a negative regulator of the action of MYB51 [94], cooperating also with MYB2 in the activation of the ABA signalling pathway in response to salt stress and water deficit [95]. MYC2 contains a JAZ interaction domain and a transcriptional activation domain which recruits the mediator of RNA polymerase II (Pol II) transcription subunit 25 (MED25) of the Mediator complex [94,96]. When the levels of JA are low, MYC2 binds JAZ proteins, leading to the repression of MYC2 activity, resulting in the suppression of JA-responsive gene expression. However, when JA concentration is elevated, JAZ repressors are degraded in the 26S proteasome and MYC2 is free to form a complex with MED25 to trigger the initiation and amplification of JA-mediated transcriptional responses [97]. *Arabidopsis* roots inoculated with *T. harzianum* showed up-regulation of *MYB51* and the *PROLINE RICH PROTEIN BstNI SUBFAMILY 1* (*PRB1*) gene involved in JA- and ET-dependent defence responses in root tissues in 4 h, and down-regulation of their expression over time, while the expression of the TF *ERF* significantly increased from 4 to 48 hpi compared to uninoculated plants [53]. This would indicate that: (i) in the first hours of interaction with *Trichoderma*, JA levels are high, in an attempt to counteract the activation of SA-dependent defences, and *MYB51* is not being negatively regulated by MYC2; and (ii) ERF4 acts as a transcriptional repressor of the SA-mediated suppression of JA-responsive genes, as SA levels would be being restored while maintaining a JA-dependent defence [98].

Via JA signalling, MYC2 also coordinates defence- and growth-related processes. In fact, in plant–*Trichoderma* interactions, MYC2 has been revealed as a master regulator of balancing the costs of plant growth and defence, since it also regulates crosstalk between the JA-dependent pathways and those of other phytohormones [12,94,99]. Of particular interest throughout these processes are, on the side of MYC2, the MED25 binding domain required for relaying JA transducing signals to the Pol II transcriptional machinery and on the MED25 side, the activator-interacting domain essential for the binding with MYC and ERF TFs [96]. The MYC2-MED25 functional complex activates JA-dependent defences but also regulates the termination of JA signalling, which means that the expression of genes dependent on the JA defence acquires an undulating shape at certain time periods [100]. MED25 has been also described as a regulator of abiotic stress responses, interacting with promoter regions of *ABA-INSENSITIVE5* (*ABI5*) TF, leading to a negative effect on ABA-regulated gene transcription [101], in the same way as histone methylation does in *WRKY40* [82]. MED25 functions as a negative regulator of primary and lateral root growth through auxin-related mechanisms [102]. MED25 cooperates with other subunits of the Mediator, as is the case with: (i) MED16, which governs the JA-ET crosstalk that also binds WRKY33 and regulates the expression of JA responsive genes and the ERF-domain TF *ORA59*; and (ii) MED18, which regulates JA-SA crosstalk and ROS production [96]. Although MED25 and MYC2 are evolutionarily conserved, their relatively weak interaction is species-specific and may explain why they are not interchangeable among plant species [103]. So, the different MYC2 and MED25 binding abilities could help explain a part of the enormous capacity of plants to alternate and progressively modify their defences to adapt to a changing environment.

Different studies have found that the application of *Trichoderma* does not produce a significant change in the expression of the *MYC2* gene [41,66] or at most a slight repression after 24 h [98]. The simplest explanation is that plants tip the balance towards growth while allowing root colonization by *Trichoderma*. However, upon attack, plants modulate MYC2-dependent defence responses that lead to a quick up-regulation when they feel attacked, either by a caterpillar herbivore [104] or by a necrotrophic fungus [105]. Conversely, *MYC2* was down-regulated in plants treated with *Trichoderma* but attacked by the coronatine-producer (hemi)biotrophic bacteria *P. syringae* pv. *tomato* strain DC3000 [47]. In this last case, plants challenged with *T. asperellum* kept similar levels of expression for the SA-dependent defence genes to the unchallenged ones, while increasing the levels of the genes involved in ET-dependent defence, providing the expected conditions for the pathogenic bacteria to be controlled. 

## 4. Long-Lasting Priming and Plant Memory

It now seems clear that with long periods of time, defences are activated by *Trichoderma* [106,107], both locally, which would limit the penetration of the fungus to the first few layers of root cortical cells [45,108], and systemically [109], which would prime the plant against the attack of different pathogens.

Systemic acquired resistance (SAR) is the fact that uninfected systemic plant parts become more resistant in response to a localized infection elsewhere in the plant. Induced systemic resistance (ISR) is a term that has emerged to define the enhanced defensive capacity of the entire plant against a broad spectrum of pathogens, acquired upon local induction by beneficial microbes [20]. Interestingly, it has recently been reported that foliar applications of *T. asperellum* were able to suppress oak powdery mildew disease in the subsequent three years [33]. It is widely accepted that SA is essential in SAR signal transduction against biotrophic pathogens while ISR was shown to be effective against attackers that are sensitive to JA/ET-dependent defences including necrotrophic pathogens and insect herbivory [20]. It is common to find in the literature that *Trichoderma* induces ISR. However, in view of the responses of plants to colonization by *Trichoderma* and the subsequent priming of defences, the terms SAR and ISR are too stiff. It has been reported that the application of *T. arundinaceum* to the seeds, and *T. harzianum* to the substrate, primed effective defences in four-week-old tomato plants at 96 hpi with *B. cinerea* [110,111]. Plants challenged with *T. arundinaceum* displayed both SA- and JA-dependent systemic defences [110]. In the case of *T. harzianum*, JA-regulated responses typically directed ISR development, although the establishment of successful systemic defence required intact JA, SA, and also ABA signalling pathways [111]. When 30-day-old tomato plants from seeds inoculated with *T. harzianum* were treated with one of its released metabolites, harzianic acid (HA), ET/JA pathways related to ISR were activated, but HA also up-regulated the SA pathway, thus causing the co-induction of ISR and SAR responses [78].

Therefore, doubts have been raised about how to precisely define the situations of priming. According to the different examples mentioned above on the undulating behaviour of the plant’s defences triggered by *Trichoderma*, it seems more accurate not to use the classic definitions of ‘SA-dependent SAR’ or ‘JA-dependent ISR’ applied to plant–*Trichoderma* interactions. As observed with the root-knot nematode (RKN) *Meloidogyne javanica* and its attack on tomato roots completing their biological cycle, *Trichoderma* seems to reprogram the plant immunity by adapting SA- and JA-dependent defences according to the pathogen infection stage [74,112]. A time course study for 144 h carried out on tomato seedlings inoculated with *T. parareesei* served to prove that reprogramming of transcription due to *Trichoderma* favours both abiotic and biotic stress responses over normal cellular functions in an undulating pattern [113]. Interestingly, the beneficial effects (lateral root development, improved defence against *B. cinerea*, and growth promotion under salt stress) were more apparent depending on proximity to the *Trichoderma* inoculation time and whether the plants were subjected to some form of stress [113].

A model based on transcription cascades involving different hormone signalling pathways has been proposed in order to explain the dynamics in plant immunity. Particularly, the changes in redox within 24 to 48 h after the activation of some kind of defence that dictate transcription pulses of different hormone signals in systemic tissues [114]. As hormone pathways are frequently antagonistic, the transcription pulses may be mutually exclusive within single cells. Nonetheless, feedforward loops may exist in which multiple hormone signals control the next transcription pulse by transcription cascading. The result is an undulating pattern of defences as the phytohormone networking provides the plant with the regulatory potential to favour, in case of need, immune response pathways over pathways that regulate normal cellular development [115]. We have seen that in *Trichoderma*–plant interactions, independently regulated TFs may function in combination with modulating key immune genes through transcription cascades. It is becoming increasingly clear that the phytohormone crosstalk also provides plant cells with the ability to launch multiple hormone-driven transcription programs without cross-interference to establish immunity or growth depending on the case [12,114].

**Figure 2:**
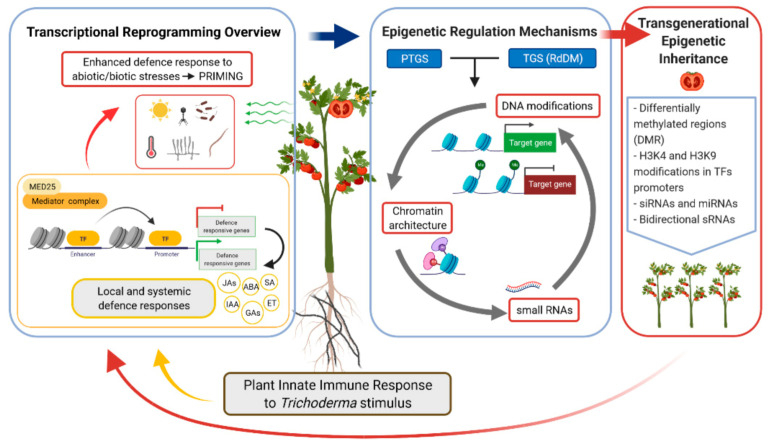
Schematic outline of plant immune local and systemic responses to *Trichoderma* priming stimulus. These responses involve a transcriptional reprogramming process where the action of enhancers (Mediator complex-subunit MED25) and transcription factors (TF) have a key role in the regulation of phytohormone synthesis pathways. Regulation of these processes is mediated by small RNAs (sRNA) through a transcriptional gene silencing (TGS) mechanism—mainly through the RNA-directed DNA methylation (RdDM) pathway—or through post-transcriptional gene silencing (PTGS), which has been proven to impact whole-genome DNA methylation patterns. The inheritance of these epigenetic marks on the offspring leads to the plant immune response against abiotic or biotic stresses without the need for an external stimulus triggered by *Trichoderma*. Abbreviations: SA, salicylic acid; JAs, jasmonates; ET, ethylene; ABA, abscisic acid; IAA, indole-3-acetic acid; GAs, gibberellins. Created in Biorender.com.

At this point, it is worth asking what happens to the stress signals when the transcription pulses disappear, or in other words when the amplitudes of the undulating curves tend to zero. It is evident that plants cannot be continuously activated under stress-free conditions, since it would be at a very high cost of energy, so signals are reverted after a prolonged decline in stress pressure [14]. The mere up-regulation of *MPK3/6* or the accumulation of phytohormones and TFs crucial for the defence of plants is not sufficient to explain the reverted effects of priming. Today, we know that signals generated after stress are stored by the plants in the form of DNA hypomethylation in the promoters of defence genes and chromatin remodelling that act as a long-lasting epigenetic immune memory [116,117]. As a continuation of the work on RKN–*Trichoderma* interaction [74], the cytosine methylation in different contexts (CpG, CHG, and CHH) of tomato plants from seeds treated and untreated with *Trichoderma* was analysed. As expected, plants treated with *Trichoderma* showed a 2.42% lower methylation profile of their genome, validating the positive plant gene regulation described in previous plant–*Trichoderma* transcriptomic studies. An overview of the processes involved in the interaction of *Trichoderma* and the plant host from the point of view of the timing through priming, epigenetic marks, and inheritance is presented in Figure 2.

Typically, histone acetylation by acetyltransferases is associated with transcriptional activation, while histone deacetylation by deacetylases (HDA) results in transcriptional repression [118]. Acetylation of H3K4 and H3K9 in the promoters of *WRKY40* and *WRKY70* agrees with higher expression in biotic defence genes [119]. Deacetylase HDA19 represses transcription of *WRKY38* and *WRKY62* and thus negatively regulates basal defence [120]. HDA19 can be induced by JA and ET, and the overexpression of the *HDA19* gene decreased the acetylation levels of H3 in *Arabidopsis* and did not increase the SA-regulated genes [121]. JAZ proteins physically interact with deacetylase HDA6 providing a resting state in which JA-dependent plant processes are repressed. It seems, therefore, that JAZ-mediated suppression of the JA-mediated transcription involves the establishment of a ‘closed’ or ‘suppressed’ chromatin state that excludes JA-responsive TFs, such as MYC2, from binding to their targets [94].

Methylation changes of histones can either increase or decrease transcription of genes [110]. Methylations at the *WRKY40* promoter negatively regulated ABA signalling and led to the activation of the SA-dependent pathway [82], while demethylase JM17 activity did not induce but rather negatively regulated dehydration stress and ABA responses by maintaining H3K4me3 methylation levels [122]. As we have already mentioned, these processes are complex and sometimes contradictory because we have seen that H3 methylations suppress but also favours the expression of defence genes [88]. Similarly, acetylation does not always match with increased gene expression, since other regulatory mechanisms, such as peroxidase production to maintain ROS homeostasis, may also be involved [113]. In any case, the most typical modifications in defence gene promoters are H3K9ac and H3K4me3 that function as molecular footprints to gene priming, and in them seems to reside an important part of the long-lasting memory. The *Arabidopsis* SA-dependent systemic priming against *Pseudomonas* was associated with H3K9ac and H3K4me3 chromatin modifications on the promoters of *WRKY6*, *WRKY29*, and *WRKY53* [87]. The mediator protein MED25 physically and functionally interacts with the acetyltransferase HAC1 which selectively regulates H3K9ac at MYC2 target promoters and thereby favours long-term JA-responsive gene expression [123]. MED25 also activates H3K9ac in the *WRKY33* promoter and is usually connected with the H3K4me3 that facilitates the expression of *WRKY70* to simultaneously regulating gene expression to fine-tune JA- and SA-dependent defence priming [86,124]. Together with MED25, MED18 also controls the balance between JA and SA, interacting with the acetyltransferase HOOKLESS1 to increase *WRKY33* expression and thereby regulating JA-SA crosstalk and ROS production [96]. As shown above, the MYC2-MED25 complex plays a fundamental role in plant–*Trichoderma* interactions, and WRKY33 and WRKY70 [40,66,72,73], as well as WRKY40 or MYB77 [72], seem to be crucial in the activation and maintenance of the priming memory. The MED25-mediated H3K4me3 and H3K9ac in promoters of *WRKY70* and *WRKY33* respectively might justify the long-lasting induction of SA and JA/ET-dependent priming in plants challenged with *Trichoderma*. Similarly, the concerted methylation/demethylation activity on H3K4me3 at the *WRKY40* promoter would explain the long-lasting down-regulation of this TF and as a result the modulation of SA- and ABA-dependent responses, balancing defence and growth. As observed in *Arabidopsis* plants, the auxin-stimulated TF *MYB77* was down-regulated at 24 hpi with *Trichoderma* [72], thus limiting the development of lateral roots. The long-lasting up-regulation of MYC2-dependent genes after a pathogen attack would agree with the concerted deacetylations on *MYB77* and *ARF* promoters [91,125] and as a result, the plant would shape auxin responses, tipping the balance towards defence when needed.

We have just seen that there are mechanisms in plant cells that store information on their behaviour during previous attacks in order to remember and adapt to new situations if required later. In addition, the plant’s responses to *Trichoderma* are effective for long-lasting priming against abiotic stresses and pathogen attacks. The energy savings provided to the plants by the priming of defences allow them to manage growth as well. In this context, the MYC2-MED25 complex, not forgetting the maintenance of ROS homeostasis, seems to play a leading role in *Trichoderma*-primed plants.

## 5. Transgenerational Inheritance

One of the focal points when addressing systemic defences maintained over time is whether priming is mediated by beneficial microbes via epigenetic mechanisms and whether they are inherited trans-generationally [20]. One could expect an affirmative answer to both questions, as there are works to show that enhanced resistance to pathogens (e.g., bacteria, fungi, or insects) in the progeny of previously attacked plants was transmitted by post-translational modifications of histones (e.g., H3K9ac, H3K27me3) at *WRKY* and defence gene promoters [126]; by DNA hypomethylation of SA-dependent defence regulatory genes [127]; and by mechanisms able to reduce DNA methylation [128]. However, it is well known that viral infections induce epigenetic changes that can also be transmitted to the progeny of infected plants [129]. As we will see below, these plants produce non-coding small RNAs (sRNA) in response to viral infection as well as to abiotic stresses that lead to hereditary changes in DNA methylation [130]. DNA methylation, controlled by both DNA methyltransferase and DNA demethylation enzymes, is an epigenetic mark that silences transposable elements (TE) and repeats. Some immune-response genes, containing repeats in their promoter regions, are negatively regulated by this mechanism. So, DNA demethylation is part of the plant immune response, potentially acting to prime the transcriptional activation of defence genes linked to TEs/repeats in systemic unchallenged tissues, including reproductive organs, thereby orchestrating transgenerational immune priming [131]. While chromatin modifications provide long-lasting priming, as DNA (de)methylation is transmitted by meiosis, it is not unreasonable to think that in this mechanism lies the transgenerational inheritance of priming.

Plants have evolved a variety of gene silencing pathways [132] to generate sRNAs that govern the expression of sequence homologous genes [133] at the transcriptional level (transcriptional gene silencing, TGS), either preventing or dampening transcription through DNA methylation and chromatin modifications, or at the post-transcriptional level (post-transcriptional gene silencing, PTGS), through RNA cleavage or translational repression. Moreover, sRNAs are key regulators that can reprogram the expression of stress- or development-related genes to balance resources between investment in development and responses to biotic and abiotic stresses [134], precisely the characteristics that *Trichoderma* confers to plants. In this regard, host cells generate sRNAs corresponding to the genome infecting pathogen. Invasive nucleic acids are transformed into double-stranded RNA and diced into primary small interfering RNAs (siRNA) through the sequential action of RNA-dependent RNA polymerases (RDR) and Dicer-like (DCL) RNase III enzymes. Then, the defence response spreads systemically throughout the plant to promote RNA silencing mediated resistance. RNA silencing is regarded as an adaptive form of antiviral immunity [135] that also has an important function in the regulation of plant endogenous processes [136]. Plants synthesize two major types of sRNAs, classified into microRNAs (miRNA) and siRNAs on the basis of their biogenesis and precursor structures. Both mature miRNAs and siRNAs associate with ARGONAUTE (AGO) proteins to guide TGS or PTGS to cognate targets based on their homology. *MIR* genes are transcribed into long single-stranded transcripts [137]. They adopt a fold-back stem-loop structure that is processed in most cases into a mature miRNA duplex by the enzyme DCL1 [135], although some miRNAs can be processed by DCL4 [138]. miRNA duplexes are methylated at their 3’ terminal nucleotide by the RNA methyltransferase HUA ENHANCER 1 (HEN1) [139,140,141] to protect them from degradation [140] and most are exported to the cytoplasm by the exportin-5 homologue HASTY (HST) [142]. One strand of the miRNA duplex acts as a guide strand and is selectively loaded onto an AGO protein to form the core of the RNA-induced complex (RISC), whereas the other strand, the passenger strand (miRNA*), is discarded from the complex and rapidly degraded. Most miRNAs associate with the protein AGO1; however, specific associations of miR408 or miR393* with AGO2, of miR390 with AGO7, and of miR165/166 with AGO10 have been reported [143,144,145,146]. Plant miRNAs promote the cleavage of their target RNA, to which they bind perfectly or near perfectly, by employing mostly AGO1 as the RNA slicer. Therefore, cleavage is assumed as the common approach for miRNA-mediated gene regulation in plants [147,148,149]. Thus, miRNAs act as signal molecules to carry information and they can accumulate to extremely high levels within plant tissues to regulate, by PTGS, the expression of target mRNAs involved in many aspects of plant biology, including growth, and defence and adaptation to stresses [18,150]. However, in addition to regulating RNA degradation, evidence exists to suggest that miRNAs with binding sites in gene promoters sometimes direct DNA methylation [151] to modulate gene expression through epigenetic modifications of the promoter or inhibit translation [152,153,154,155,156,157].

Some pathogens and pests deliver sRNAs into plant host cells to act as effectors through a mechanism that silences host genes in order to suppress host immunity and achieve infection. Conversely, sRNAs of the host travel into the attacker cells to inhibit their virulence. This bidirectional sRNA traffic is known as ‘cross-kingdom RNA interference (RNAi)’ [158]. The pathogenic fungus *B. cinerea* delivers several sRNAs into host plant cells during infection [159]. Some of these fungal sRNAs have complementarity to host immunity genes (e.g., *MPK1/2*) that are down-regulated during *B. cinerea* infection by hijacking host sRNAs machinery with its own set of sRNAs [159]. Another example of sRNAs delivered into the host cells is the case of miRNA-like RNA1 (Pst-milR1) produced by *Puccinia striiformis* (*Pst*), one of the most destructive pathogens of wheat. Pst-milR1 suppresses wheat defences during *Pst* infection by targeting the *PR-2* gene to impair wheat resistance to *Pst* [160]. An example of the parasite-to-host transfer of sRNAs comes from the parasitic plant *Cuscuta campestris,* which produces many miRNAs, some of them specifically targeting host mRNAs involved in defence [161]. Similarly, it has been suggested that *Trichoderma* could be using sRNAs as effector molecules that utilize the host RNA silencing machinery to establish a symbiotic relationship with *Arabidopsis* by binding to AGO1 for silencing host genes involved in plant immunity [23].

It is therefore to be expected that somehow some of the *Trichoderma* effects could also be inherited by the plant. To confirm this assumption, it would be necessary to explore: (i) what characteristics *Trichoderma* confers on the plant that are non-genetically transmitted to the offspring; and (ii) whether DNA demethylation and/or methylation occurs and/or sRNA production is increased in the next generation of plants.

During the study in which we described that *T. atroviride* was able to colonize tomato roots by making the plant adapt its systemic SA- and JA-dependent defences according to the RKN *M. javanica* infection stage, we also observed that the first generation (F_1_) of *T. atroviride*-primed tomato plants inherited resistance to RKN [74]. This defence priming inheritance by the offspring varied depending on whether the F_1_ plants came from seeds of plants treated with *T. atroviride*, *M. javanica*, or both arranged in different pots following a split-root approach [162]. In this way, F_1_ plants derived from *T. atroviride* + *M. javanica* split-rooted plants exhibited a dramatic reduction in size, but in contrast they had the SA- and JA-dependent defences activated, expressing even stronger defence priming than offspring of unprimed plants. Instead, F_1_ plants derived from plants that were only treated with *T. atroviride* showed the largest sizes and highest green mass values. Thus, plant growth promotion induced by *T. atroviride* would also be inherited and we would expect them to be poorly defended. However, these F_1_ plants displayed active defence when they were infected with *M. javanica*, with very low levels of SA and JA but with increased auxin-induced ROS production, which was sufficient to protect the plants from a subsequent RKN attack. This means that the progeny of *T. atroviride*-primed plants would be displaying increased size and resistance to *M. javanica* without fitness costs. Seen as a whole, the results of that study indicated that the *Trichoderma* switching signatures involved in balancing the plant’s defence and growth would be inherited in a *T. atroviride* treatment conditions-dependent manner [74].

The alternative defence mechanism based on auxin-induced ROS production in plants that choose to grow, but not at the cost of being left unprotected, is compatible with the *Trichoderma* ability to promote more and longer secondary root hairs in cucumber plants, increasing the total absorptive surface and facilitating the uptake of nutrients, resulting in increased plant biomass [163]. A recent study [164] has proposed a molecular mechanism by which endogenous auxin activates several *ARFs* to up-regulate the expression of the TF *RSL4* gene involved in ROS-mediated regulation of root hair elongation, by promoting the expression and activity of NADPH oxidase and peroxidase proteins. In this case, the MYC2-dependent auxin signalling pathway [94] and the interaction between MYB77 and ARFs [125] would be a gene regulation backup, acting as a major defence strategy during plant growth.

The bidirectional sRNAs study between the plants and *Trichoderma* is a hitherto unexplored field of research that will need further attention. As far as guide miRNAs are concerned, it would be very extensive to list their different functions as they act as integrators of multiple environmental cues. As a general rule, components of the plant immune system are constitutively repressed by specific miRNAs in non-infected tissues, but their down-regulation around an infected zone activates defence-related genes to restrict fungal growth [18]. Although it has also been noted that the activation of some miRNAs may also contribute to suppressing certain defence suppressors, providing some sort of immunity [18]. Just to mention one example, we have found that several miRNAs targeted to genes related to auxin, root growth, and defence were up-regulated in *T. atroviride*–tomato plants. Particularly, the transcription of *MIR393* and *MIR160* was induced upon *Trichoderma* perception by the plants. miR393 increases tolerance to ROS and targets the auxin receptors TIR1 to inhibit auxin signalling—which might be required for root growth regulation in response to stress—and alleviates its antagonism on SA signalling [165]. miR160 has a role in maintaining proper auxin homeostasis, control of root cap formation, and the regulation of *ARF16* expression required to maintain the normal growth and development of aerial organs and lateral root production [166,167].

It has been proven that DNA methylation of regions flanking the miRNA coding sequence affects miRNA biogenesis [168] and the production of miRNAs targeting a promoter region along with an increase in the DNA methylation level are accompanied by the decreased expression of the corresponding gene. As a result, the ensuing silent state is inherited in the following generation. Therefore, further research is needed to clarify the basic molecular mechanisms involved in the inheritance of the epigenome triggered by the *Trichoderma*-induced plant priming responses.

## 6. Conclusions

*Trichoderma* is a common resident of the rhizosphere where it first evolved as a mycoparasite and then established symbiotic relationships with plants, becoming an endophyte. A fine-tuned crosstalk with the plants has allowed many strains of *Trichoderma* to carve out an advantageous ecological niche while providing benefits to their hosts. There are a growing number of reports that describe how *Trichoderma* strains try to achieve their goals and we can be faced with results that may be contradictory, depending on the timing and conditions of each *Trichoderma*–plant interaction study. It is now clear that time-course studies are the most appropriate to draw conclusions, that plant responses to *Trichoderma* signals follow undulating dynamics, that plants store the *Trichoderma* signature in their memory and these signatures can be passed on to their offspring. The beneficial action that *Trichoderma* has on plants is modulated by different molecular hubs that condition the immediate and long-lasting systemic responses, orchestrating the trade-off between plant growth and defence, as we have seen with the acceleration and moderation of MPK3/6 cascades; ROS homeostasis; *WRKY4*, *WRKY18*, *WRKY33*, *WRKY40*, *WRKY70*, *MYB15*, *MYB51*, *MYB72*, *MYB77*, *MIR160*, or *MIR393* transcription dynamics; histone acetylation and methylation at the *WRKY33*, *WRKY40*, or *WRKY70* promoters; the MYC2-MED25 complex activation; and the yet to be further explored DNA (hypo)methylation and ‘cross-kingdom RNAi’ between *Trichoderma* and plants. Moreover, the discovery that the *Trichoderma* switching signatures involved in balancing the plant’s defence and growth can be inherited opens up new possibilities for the biotechnological application of this fungus. A first consequence may be the registration of *Trichoderma* strains for commercial use in agriculture, either as plant protection products inducing systemic resistance or as biostimulants, since it seems misguided to attribute to *Trichoderma* a trait that depends on plant decision-making.

In terms of future prospects, the bidirectional traffic of sRNA between *Trichoderma* and plants has a role to play in understanding plant responses to *Trichoderma* priming and its heritability. It would be very interesting to explore whether and to what extent the *Trichoderma* signature conferred on transgenic plants is also heritable or even transmissible through grafting. This will allow us to know whether such a signature is the result of a whole or whether, on the contrary, it may be a functionally compartmentalized effect. 

## Figures and Tables

**Table 1:** Summary of plant WRKY, MYB, and MYC transcription factors (TF) regulated by *Trichoderma* spp. interactions through mitogen-activated protein kinases (MAPK) or Ca^2+^-dependent protein kinase (CDPK) cascades and their biological effects on plant hosts.

TFs	Biological Process ^1^	*Trichoderma* Strain	Plant Host	Regulatory Effect	Reference
**WRKY2**	Abscisic acid (ABA)-mediated responses, establishment of cell polarity and pollen development	*T. harzianum* M10	Tomato Micro-Tom	Up-regulated (Up) after *T. harzianum* treatment. Down-regulated (Down) after *T. harzianum* + *Rhizoctonia solani* application	[78] ^2^
**WRKY4**	Negative regulation of jasmonic acid (JA)-, ethylene (ET)-, and salicylic acid (SA)-dependent defence responses	*T. erinaceum* T7	Tomato S-22	Down after *T. erinaceum* and *T. erinaceum* + *Fusarium oxysporum* f. sp. *lycopersici* (FOL) application at 24 h and 48 h (in roots) and 48 h in leaves	[50]
**WRKY8**	Positive regulator of fungal attack, activated by ABA, salt stress, wounding, and H_2_O_2_	*T. atroviride* IMI206040	*Arabidopsis* Col-0	Up after *T. atroviride* treatment at 24–72 h	[73]
**WRKY18**	Negative regulator of SA-dependent defence responses, positive regulator of JA- dependent defence responses to biotic stress, and ABA-dependent defence response to abiotic stress	*T. asperelloides* T203	*Arabidopsis* Col-0	Up after *T. asperelloides* treatment at 9–24 h. Enables root colonization	[72] ^2^
**WRKY31**	Positive regulation of JA-, SA-, gibberellins (GA)-dependent defence responses and terpene biosynthesis	*T. erinaceum* T7	Tomato S-22	Up after *T. erinaceum* and *T. erinaceum* + FOL at 24 h and 48 h (in roots and leaves)	[50]
**WRKY33**	Negative regulation of SA-dependent early defence responses and positive regulation of JA-dependent defence responses at later stages. Activation and maintenance of the priming memory	*T. atroviride* IMI206040	*Arabidopsis* Col-0	Up after *T. atroviride* treatment, at 48–72 h	[79]
		*T. velutinum* T028	Bean Canela	Up after *T. velutinum* treatment. Down after *T. velutinum* + *R. solani* application	[66]
		*T. atroviride* IMI206040	*Arabidopsis* Col-0	Down after *T. atroviride* treatment at 24–72 h	[73]
**WRKY37**	Defence response to fungal attack	*T. erinaceum* T7	Tomato S-22	Up after *T. erinaceum* and *T. erinaceum* + FOL application at 24 h (in roots and leaves)	[50]
**WRKY38**	Negative regulator of SA-dependent defence responses, susceptible to inactivation by JA-induced histone deacetylase HDA19	*T. atroviride* IMI206040	*Arabidopsis* Col-0	Up after *T. atroviride* treatment at 96 and 144 h	[73]
**WRKY40**	Central negative regulation of ABA signalling, positive regulation of JA-dependent defence responses. Activation and maintenance of the priming memory	*T. asperelloides* T203	*Arabidopsis* Col-0	Up after *T. asperelloides* treatment at 9, 24, and 96 h. Enables root colonization	[72] ^2^
**WRKY54**	Negative regulator of SA biosynthesis, positive regulator of SA-dependent defence responses and brassinosteroids (BR)-regulated plant growth and drought responses	*T. atroviride* IMI206040	*Arabidopsis* Col-0	Down after *T. atroviride* treatment, at 24–48 h	[73]
		*T. harzianum* CECT2413 (T34)	*Arabidopsis* Col-0	Down after *T. harzianum* treatment in the first 24 h of interaction	[40]
**WRKY55**	Positive regulator of reactive oxygen species (ROS) production and SA-dependent defence responses	*T. atroviride* P1	Tomato San Marzano nano	Up after *T. atroviride* treatment at late stages of defence	[80] ^2^
**WRKY60**	Negative regulator of SA-dependent defence responses, positive regulator of JA- dependent defence responses to biotic stress and ABA-dependent defence response to abiotic stress	*T. atroviride* IMI206040	*Arabidopsis* Col-0	Down after *T. atroviride* treatment at 24 h, and up at 144 h	[73]
**WRKY70**	Positive regulator of SA-dependent defence responses and negative regulator of JA-inducible genes, acting as a node of convergence for defence signals. Activation and maintenance of the priming memory	*T. atroviride* IMI206040	*Arabidopsis* Col-0	Down after *T. atroviride* treatment at 24 and 48 h, and up at 144 h	[73]
**WRKY78**	Positive regulator of plant size and development	*T. harzianum* M10	Tomato Micro-Tom	Down after *T. harzianum* + *R. solani* application at late stages	[78] ^2^
**MYB51**	Positive regulator of indole glucosinolate biosynthesis and shikimate pathway in response to biotic and abiotic stress	*T. asperelloides* T203	*Arabidopsis* Col-0	Up in control and salt-stressed plants after *T. asperelloides* treatment at 9, 24, and 48 h	[72] ^2^
		*T. asperellum* IsmT5	*Arabidopsis* Col-0	Up after exposure to *T. asperellum* volatiles	[81]
		*T. harzianum* T78	*Arabidopsis* Col-0	Up after *T. atroviride* treatment in only 4 h and down over time	[53]
**MYB68**	Positive regulator of root development and hardening, and defence responses to abiotic stresses	*T. atroviride* P1	Tomato San Marzano nano	Up after *T. atroviride* treatment at late stages of being challenged with aphids	[80] ^2^
**MYB72**	Positive regulator of JA/ET-dependent defence responses and callose accumulation	*T. asperellum* T34	*Arabidopsis* Col-0	Up after *T. asperellum* treatment	[70]
		*T. asperelloides* T203	*Arabidopsis* Col-0	Up after *T. asperelloides* treatment at 24 h	[72] ^2^
**MYB77**	Positive regulator of lateral root development and auxin-dependent responses. Activation and maintenance of the priming memory	*T. asperelloides* T203	*Arabidopsis* Col-0	Up after *T. asperelloides* treatment at 9, 24, and 48 h	[72] ^2^
**MYC2**	Regulatory hub within the JA signalling pathway to balance the plant’s growth and defence responses. Negative regulator of MYB51 action. Positive regulator of lateral root growth, ROS, and ABA-dependent responses. Activation and maintenance of the priming memory	*T. parareesei* IMI113135 (T6), *T. asperellum* IMI296237 (T25), *T. harzianum* CECT2413 (T34)	Tomato Marmande	Down after *Trichoderma* spp. + *Pseudomonas syringae* DC3000 application	[47]
		*T. atroviride* IMI352941 (T11)	Tomato Marmande	Down in the offspring of plants primed with *T. atroviride* and *T. atroviride* + nematode application	[74]

^1^ Generic Gene Ontology (GO) biological process for the homologous TF annotated at the *Arabidopsis* Information Resource (TAIR) database (https://www.arabidopsis.org/index.jsp); ^2^ These research studies are based on high-throughput data analysis and describe additional TFs to those presented in this table.

## Data Availability

Data is contained within the article.
